# A commentary on ‘Salvage surgeries for splanchnic artery aneurysms after failed endovascular therapy: case series’

**DOI:** 10.1097/JS9.0000000000001206

**Published:** 2024-03-04

**Authors:** Long Yu, Qian Xia, Feng Su, Bin Song, Liwei Zhang

**Affiliations:** Department of Interventional Vascular Surgery, General Hospital of Northern Theater Command, Shenyang, Liaoning, People’s Republic of China

*Dear Editor*,Visceral artery aneurysms (VAAs) are an uncommon and potentially fatal vascular condition that mostly affects the splenic artery but can also affect the hepatic and celiac arteries. Incidence of VAAs in the general population ranges from 0.1 to 2%^1,2^. Lin *et al*.^3^ conducted a retrospective assessment of patients who underwent salvage procedures for splanchnic artery aneurysms after unsuccessful endovascular therapy. In cases when endovascular therapy fails to control splanchnic aneurysms, surgical surgery appears to be a practical, safe, and successful option.

This retrospective case series includes 5 of 73 patients from 2019 to 2022. For splanchnic aneurysms, 73 patients underwent endovascular therapy; 13 endovascular trials were unsuccessful; and 5 patients had salvage operations. According to the study’s results, coil migration, a persisting mass effect from the post-embolized aneurysm, a lack of room for properly placing the covered stent, or an inability to cannulate a catheter were the main reasons why endovascular therapy failed. All patients experienced symptom relief, and the mean length of hospital stay was 9 days (mean±SD, 8.8±1.6 days). Not a single patient experienced 90-day postoperative morbidity or death. One patient experienced a modest residual asymptomatic celiac artery aneurysm (8 mm in diameter) over the follow-up period (mean±SD, 24±10 months), for which conservative treatment was administered because of underlying liver cirrhosis.

The study also clarifies the suggestion of a flowchart for patients’ decision-making in the event that endovascular trials for splanchnic aneurysms are unsuccessful. Analysis findings, if the aneurysm is located outside of a solid organ and originates from non-expendable feeding vessels, the options for salvage surgery are as follows: partial aneurysmectomy with direct closure of the feeding vessel orifice, aneurysmectomy with vascular reconstruction, and aneurysmectomy along with the involved organ if the aneurysm is located within the parenchyma of solid organs. This customized flowchart could have a big impact on clinical practice in the future. Still, there were a lot of limitations. Firstly, it’s possible that some residual confounding variables were overlooked. Secondly, there can be bias because this is a retrospective study. Lastly, the follow-up is insufficient to assess the long-term rates of operation^4^.

To verify this result, deeper and multicenter research is needed. To determine the optimal course of treatment, further in-depth prospective research, clinical trials, and analysis of indications and risk factors in different patient subgroups are needed. The following elements can be included in future research designs: First, do a complete multicenter study to validate current findings and look at risk factors and indications in additional patient subgroups. Second, larger patient populations in prospective studies, well-defined and standardized imaging examinations, and uniform clinical and imaging follow-up and management are needed^5^. Finally, reasonable inclusion and exclusion criteria are needed to make the results applicable to all patient populations.

## Ethical approval

Not applicable.

## Consent

Not applicable.

## Source of funding

Not applicable.

## Author contribution

L.Y.: study concept or design, data collection, data analysis or interpretation, and writing the paper; Q.X.: data collection; F.S.: data analysis or interpretation; B.S.: data analysis or interpretation; L.Z.: study concept or design, writing, and revising the paper.

## Conflicts of interest disclosure

This manuscript is a comment without conflicts of interest.

## Research registration unique identifying number (UIN)

Not applicable.

## Guarantor

Liwei Zhang.

## Data availability statement

This manuscript is a comment. Don’t need a Data availability statement. However, all the data from the current study are publicly available.

## Provenance and peer review

This manuscript is a comment without being invited.
